# Case Report: Accessible Digital Musical Instrument Can Be Used for Active Music Therapy in a Person With Severe Dementia and Worsening Behavioral and Psychological Symptoms: A Case Study Over a Year and a Half

**DOI:** 10.3389/fneur.2022.831523

**Published:** 2022-04-06

**Authors:** Eisuke Kondo, Ken-ichi Tabei, Ryuhei Okuno, Kenzo Akazawa

**Affiliations:** ^1^Nursing Care Health Facility Asahina, Medical Corporation Nakamurakai, Kanagawa, Japan; ^2^School of Industrial Technology, Advanced Institute of Industrial Technology, Tokyo Metropolitan Public University Corporation, Tokyo, Japan; ^3^Department of Neurology, Mie University, Tsu, Japan; ^4^Department of Electrical and Electronic Engineering, Setsunan University, Osaka, Japan; ^5^Advanced Applied Music Institute, Social Welfare Organization Kibounoie, Hyogo, Japan

**Keywords:** dementia, accessible digital musical instrument, cyber musical instrument with score, neuropsychological test, behavioral and psychological symptoms of dementia

## Abstract

Despite the fact that accessible digital musical instruments can take into account the level of cognitive demands, previous studies have been conducted with patients with mild cognitive impairment (MCI), and it is not known whether they can be used by people with moderate to severe dementia or dementia with worsening behavioral and psychological symptoms of dementia (BPSD). The participant was an 88-year-old woman with vascular dementia (VaD) (Mini-Mental State Examination [MMSE] and Neuropsychiatric Inventory [NPI] scores: 8 and 20, respectively). Music therapy (MT) was provided twice a week for 15 min, and MT sessions spanned over 18 months. For the MT, we used the cyber musical instrument with score (Cymis), an accessible digital musical instrument; it could be played using a touch panel and switches. The cognitive function of the participant declined further, with MMSE scores of 4 after 1 year and 0 after 1.5 years. BPSD peaked with the NPI score of 54 at 1 year and declined thereafter, although only apathy remained. Despite these changes, during MT, she was able to play the accessible digital musical instrument and focus on the performance. These results suggest that even patients with severe VaD can play an accessible digital instrument and continue active music therapy even if their BPSD progress with cognitive decline.

## Introduction

To date, no fundamental cure has been developed for many forms of dementia (1). In addition to pharmacological treatment with anti-dementia drugs that slow down the progression of symptoms, music therapy (MT) is widely used in the treatment of dementia (2–6). Musical instrument playing involves motor and cognitive functions simultaneously (7). Because of this multimodal intervention, musical instruments are often used in MT for dementia (8–15).

In healthy elderly people and people with mild cognitive impairment (MCI), most interventions use pianos (16, 17), although in dementia, percussion is the main musical instrument type (12, 18). In addition to rhythm, music has elements of melody and harmony. It is very disadvantageous to be unable to play melodic and harmonic elements as cognitive function declines. Accessible digital musical instruments compensate for this disadvantage. Current advances in music technology have enabled the creation of customized accessible digital musical instruments, and in recent years, the use of music technology in music therapy has been gaining attention (19). Han et al. (20) aimed to evaluate the effect of a cognitive intervention with an accessible digital musical instrument on MCI. In this prospective study, 24 patients with MCI (intervention group, 12; and control group, 12) were enrolled. An electronic device with musical instruments and a song-based cognitive stimulation therapy protocol was developed for the intervention group. In the intervention group, the Mini-Mental State Examination (MMSE) and Montreal Cognitive Assessment scores improved significantly after the 10-week intervention.

Despite the fact that accessible digital musical instruments can take into account the level of cognitive demands, previous studies have been conducted with patients with MCI, and it is not known whether such methods can be used by people with moderate to severe dementia or dementia with worsening behavioral and psychological symptoms of dementia (BPSD). We asked patients with severe dementia to use the accessible digital musical instrument Cymis (cyber musical instrument with score) (21), which has already been used in MT for patients with cerebral palsy, to assess whether they could play it when their cognitive function declined or when their BPSD worsened.

We report a case study of a patient with severe vascular dementia (VaD) who was able to play an accessible digital instrument and to continue active MT even as her cognitive function declined and her BPSD progressed over an 18-month period.

## Patient Information

### Clinical Findings, Timeline, and Diagnostic Assessment

**Figure 1:**
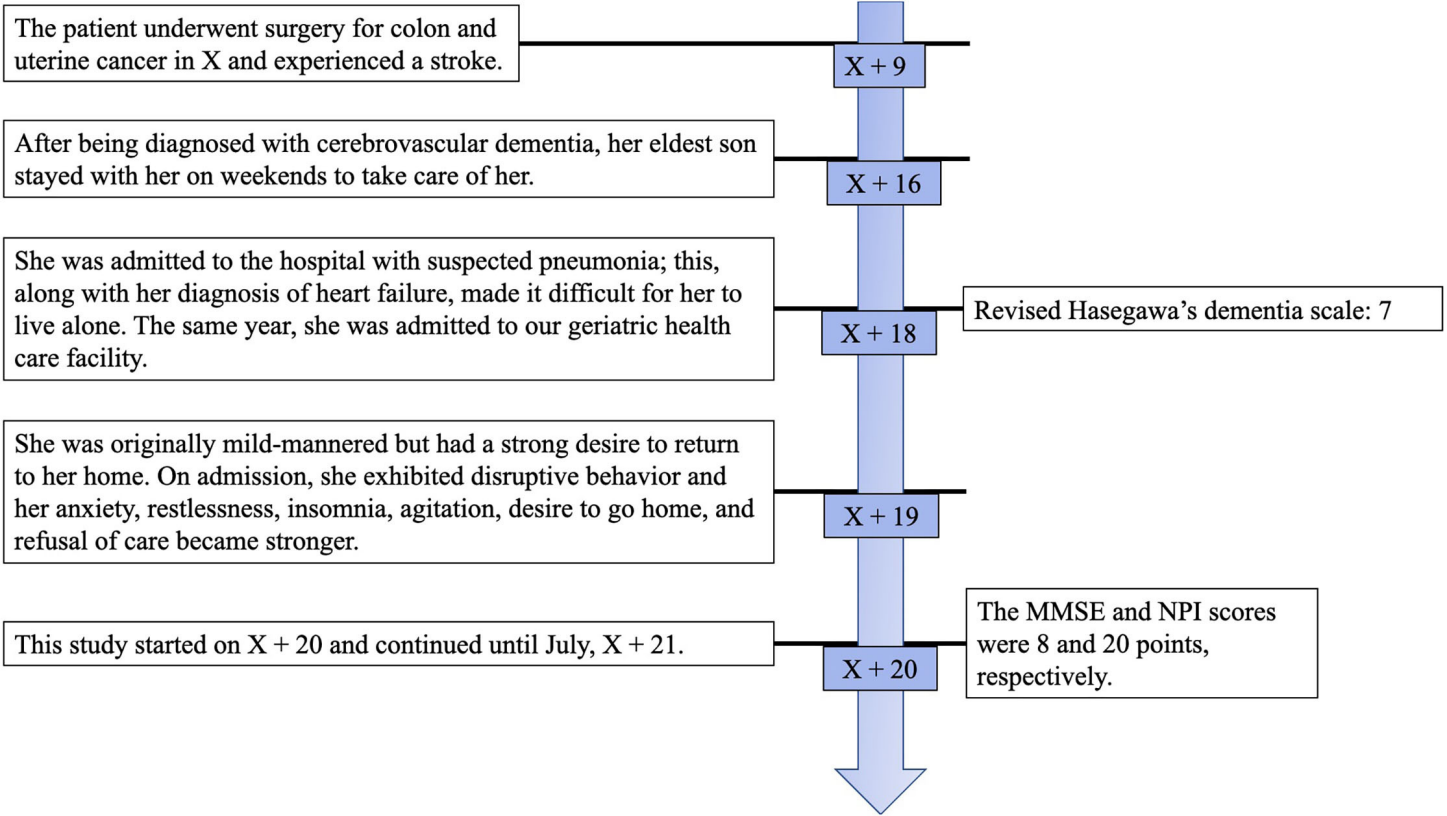
Clinical findings, timeline.

Ms. A, an 88-year-old right-handed Japanese woman was born in North Korea. After World War II, she moved to Japan. She worked as an office worker at a clothing company and learned calligraphy and the koto (Japanese harp) as a child. Ms. A has a son and a daughter from her marriage. She underwent surgery for colon and uterine cancer in X and had a stroke in X + 9. After losing her husband in X + 11, she lived alone. After being diagnosed with cerebrovascular dementia in X + 16, her eldest son stayed with her on weekends to take care of her. Ms. A used day care five times a week and nursing helpers five times a week in the mornings and evenings. In October, X + 18, she was admitted to the hospital with suspected pneumonia; this, along with her diagnosis of heart failure, made it difficult for her to live alone, and in November of the same year, she was admitted to our geriatric health care facility. At the time of admission to the facility, she had heart disease and was given medication for her hypertension. She received a score of 7 on the revised Hasegawa's dementia scale (22), with point scores of 0 for time disorientation, 1 for place disorientation, 3 for word recognition, and 3 for object recognition. She was originally mild-mannered but had a strong desire to return to her home and exhibited disruptive behavior on admission; after September, X + 19, her anxiety, restlessness, insomnia, agitation, desire to go home, and refusal of care became stronger. She participated in group MT several times but was not actively involved. During the day, Ms. A spent most of her time asleep and did not interact with others. In the afternoons, she exhibited stronger BPSD; she would cry from anxiety and clap her hands for attention. This study started in January, X + 20 and continued until July, X + 21. It was approved by the Research Safety and Ethics Committee of AIIT. Informed consent was given by the music therapist and by Ms. A and her care staff at the health care facility. On admission, Ms. A's score for the MMSE was 8 and that for the Neuropsychiatric Inventory (NPI) was 20. The caregiver completed an assessment of Ms. A's BPSD status. All tests were performed at the geriatric health care facility where Ms. A was admitted (Figure 1).

### Therapeutic Intervention

#### Neuropsychological Tests

Evaluation was conducted using the MMSE (23) and NPI (24). The MMSE was used to quantify intellectual function, and the NPI was used to assess the 10 behavioral disturbances that occur in patients with dementia: delusions, hallucinations, dysphoria, anxiety, agitation/aggression, euphoria, disinhibition, irritability/lability, apathy, and aberrant motor activity. The NPI uses a screening strategy to minimize administration time by examining and scoring only those behavioral domains with responses to screening questions. The frequency and severity of each behavior were determined. Information for the NPI was obtained from a caregiver familiar with the patient's behavior. Neuropsychological tests were conducted before the intervention, and at 6, 12, and 18 months after the intervention to compare the results. Each value was statistically processed using a chi-squared test.

#### Playing an Accessible Digital Musical Instrument

**Figure 2:**
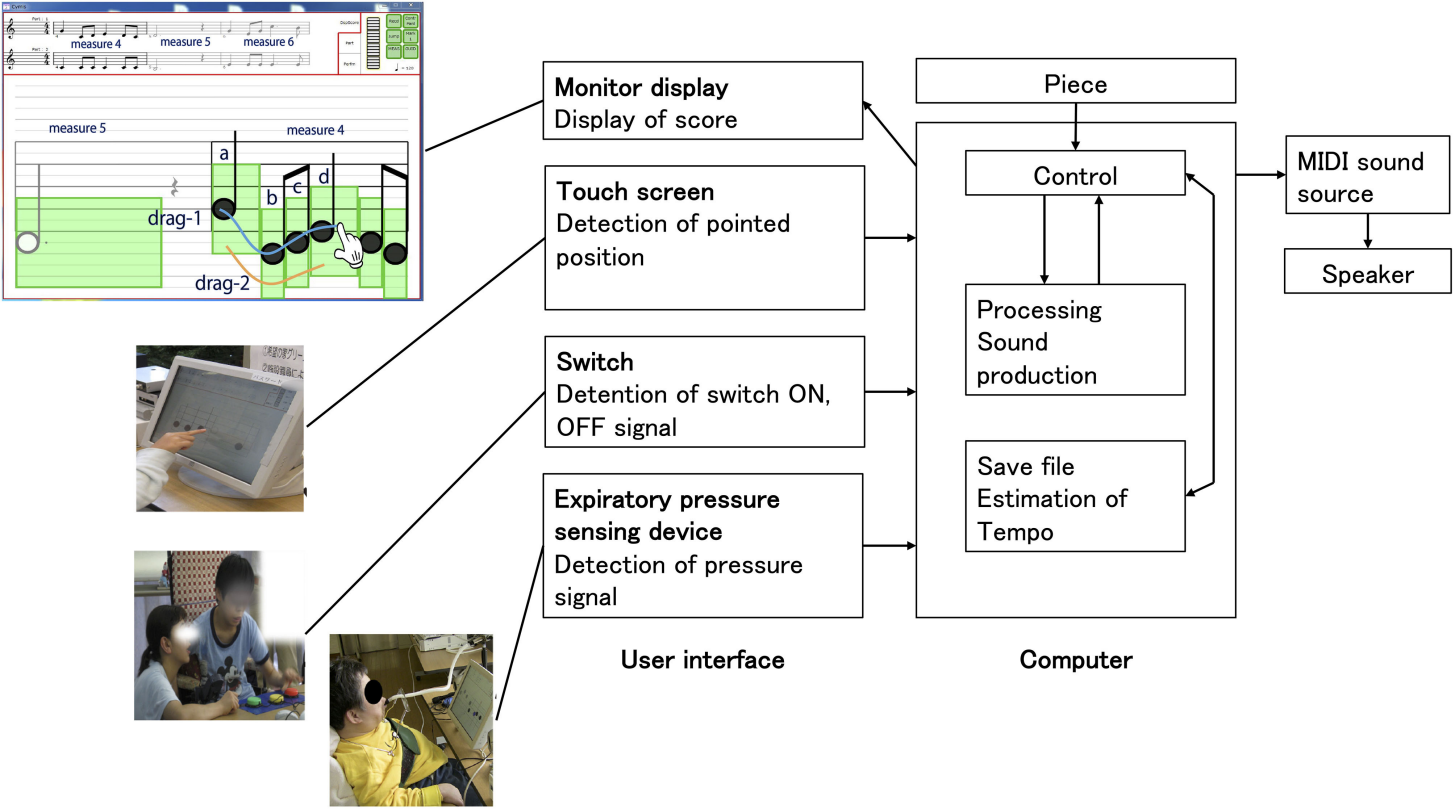
For the musical instrumental performance, we used an accessible digital musical instrument called the Cymis (cyber musical instrument with score) [Akazawa et al. (21)], which has a built-in programmed score and is played using touch panels and switches. The technical novelty of the Cymis lies in its human interface devices that emphasize the sensory experience and is characterized by its built-in score information and program. This computerized musical instrument system comprises a monitor display, a personal computer, a sound source, a speaker, and human interface devices (image sensor for finger movement detection, touch panel, and others). The system is an electronic musical instrument that is barrier-free, allows understanding of music notation, and allows beginners of any age to easily play music comprising melody, rhythm, and harmony.

Individual sessions for MT were held twice a week, for 15 min per session in the evening, over 18 months. For the musical instrumental performance, we used an accessible digital musical instrument called the Cymis (21), which has a built-in programmed score and is played using touch panels and switches (Figure 2). The technical novelty of the Cymis lies in its human interface devices that emphasize the sensory experience and is characterized by its built-in score information and program. This computerized musical instrument system comprises a monitor display, a personal computer, a sound source, a speaker, and human interface devices (image sensor for finger movement detection, touch panel, and others). The system is an electronic musical instrument that is barrier-free, allows for the understanding of music notation, and allows beginners of any age to easily play music comprising melody, rhythm, and harmony. As Ms. A was often in an anxious state of mind, we used “Kojo no Tsuki,” which is a song in the minor key, to calm her anxiety. The melody of “Kojo no Tsuki” was familiar to Ms. A; it comprises a series of quarter notes every beat, and the rhythm is consistent, making it easy to play on the Cymis by using the image sensor for finger movement detection and touch panel. There was no change in this procedure or the music chosen for the session.

## Follow-Up Period and Outcomes

**Figure 3:**
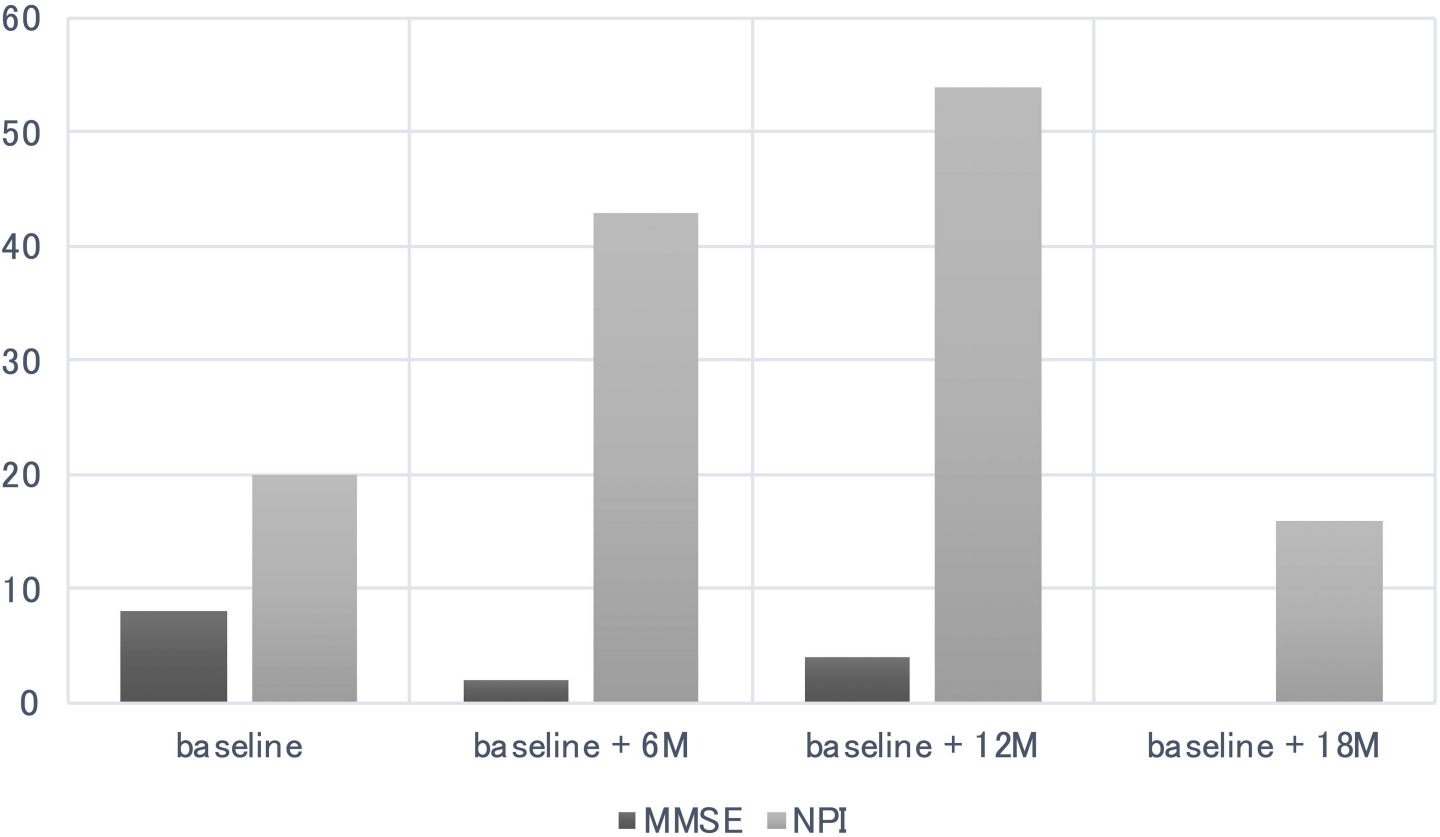
Cognitive function of the participant declined further compared to pre-intervention, with a Mini-Mental State Examination (MMSE) scores of 4 after 1 year (*p* = 0.388), behavioral and psychological symptoms of dementia (BPSD) peaked with the NPI score of 54 after 1 year (*p* < 0.001). After a year and half, Ms. A's MMSE score was 0 (*p* < 0.001) and Neuropsychiatric Inventory (NPI) was 16 (*p* = 0.617), but only apathy remained.

The cognitive function of the participant declined further compared with that pre-intervention, with an MMSE score of 4 after 1 year (*p* = 0.388), and BPSD peaked with an NPI score of 54 after 1 year (*p* < 0.001). After a year and half, Ms. A's MMSE score was 0 (*p* < 0.001) and her NPI score was 16 (*p* = 0.617), although only apathy remained (Figure 3, Table 1).

**Table 1:** All examined items from which the NPI score was calculated.

	**Delusion**	**Hallucinations**	**Agitation**	**Anxiety**	**Dysphoria**	**Euphoria**	**Apathy**	**Disinhibition**	**Irritability**	**Aberrant motor**	**Total**
										**behavior**	
baseline	0	0	4	4	2	0	2	0	0	8	20
baseline + 6M	0	0	3	6	12	0	4	4	6	8	43
baseline + 12M	0	0	4	9	12	0	6	2	9	12	54
baseline + 18M	0	0	0	1	2	0	12	0	1	0	16

*The cognitive function of the patient further declined compared with that pre-intervention, BPSD peaked, and NPI score was calculated to be 54 points after 1 year (p < 0.001). After 1.5 years, the NPI score was 16 points (p = 0.617), although only apathy remained*.

*BPSD, behavioral and psychological symptoms of dementia; NPI, neuropsychiatric inventory*.

Despite her cognitive and behavioral abnormalities, the patient continued to play the Cymis twice a week for 15 min per session over an 18-month period. During the MT, Ms. A was able to play the Cymis and focused on the performance even when she was restless. After playing, Ms. A showed a sense of accomplishment and familiarity with the musical instrument. Her music therapist considered her an excellent player with advanced skills in accuracy, rhythm, and tempo.

Sessions (S) S1 to S17 dealt with using the touch panel (playing notes), and S18 onward dealt with using one switch (playing beats), and subsequently, the touch panel (playing notes or beats). In S24, although the patient was very unsettled, she understood that she could play notes by touching the rectangular part of the musical instrument and played it with her thumb; however, she subsequently returned to playing the musical instrument with her index finger to make it easier for her to play. In S4, Ms. A remembered having played the musical instrument previously. From S15 onward, the Ms. A's cognitive and verbal functions gradually declined, and she had difficulty recalling words and repeated a single word. Ms. A's mental decline progressed, and she had difficulty in writing her name and letters. Ms. A would cry due to restlessness and anxiety. However, Ms. A was able to turn her attention to her performance while playing the Cymis. It was observed that Ms. A would hit the touch panel and switches to play the musical instrument when she was restless. She would also repeatedly press the switch when the sound did not play due to an operational error. After the performance, a sense of accomplishment, positive feedback, and a sense of familiarity were observed in the patient: she would say “It felt good,” “It was easy,” and “I've done it before.” In the self-evaluation, although Ms. A was unable to write, she welcomed the opportunity to self-evaluate, and although she was unable to say words due to the decline in her language function around S74, she expressed her anxiety and smiled when we listened to her. After S74, her language function declined and she could not speak, although when we talked to her, she expressed her anxiety and smiled. After S84, her activities of daily living (ADL) decreased and she was confined to a wheelchair, and her hand movements and upper arm range of motion also decreased. However, she was able to operate switches and touch panels with only simple support for the MT. After S90, neuropsychological tests were not performed. After S120, the patient's ADL decreased further, and she became unsteady in a seated position and was sometimes unresponsive. She became unable to understand the operation of the switches and could not operate them, saying “I don't know.” At present, the MT supports Ms. A's hand to operate the touch panel, and she is able to grasp the rectangle and follow the order of the notes with her fingers and press them to play. After the performance, she smiles and applauds.

## Discussion

Despite the fact that accessible digital musical instruments can take into account the level of cognitive demands, previous studies (20) have been conducted with MCI, and it is not known whether these instuments can be used by people with moderate to severe dementia or dementia with worsening BPSD. In our case, the patient with VaD was able to play the Cymis throughout the 18 months of MT. The patient's cognitive function of the participant declined further, and BPSD peaked, until only apathy remained. Despite these changes, during MT, the patient was able to play the accessible digital musical instrument and to focus on the performance. These results suggest that patients with severe VaD can play an accessible digital instrument and continue active MT even if their BPSD progress with cognitive decline.

The relationship between the exacerbation of BPSD with cognitive decline and ability to play musical instruments, which plays an important role in active MT, remains unknown. Herein, we report a case of long-term individual MT using an accessible digital musical instrument, which resulted in the person being able to play the musical instrument and to focus on the performance even when she was restless. The Cymis, which has a built-in programmed score, is played using a touch panel and switches. MT gave the patient a sense of accomplishment and familiarity.

The patient in this study learned to play an accessible digital musical instrument for the first time in the late stage of VaD and continued to play it despite progressive BPSD with cognitive decline. These findings suggest that it is possible to learn to play a new accessible digital musical instrument even in the late stage of VaD. Previous case reports have described several patients with dementia who continued to skillfully play musical instruments; however, these patients were professional musicians premorbidly and had a preserved musical ability even after the diagnosis of dementia (25–27). In contrast, Ms. A. had never played a musical instrument other than the koto before her diagnosis of VaD. To the best of our knowledge, this is the first reported case of a person who was able to play a new accessible digital musical instrument in the late stage of VaD, despite no prior professional musical training. There may be several reasons for this. First, playing a musical instrument requires different circuits from those essential to BPSD. Up to 23 brain structures were associated with an increased risk of developing BPSD (28), for which the frontal volume was the most powerful predictor of the frontal gyri, anterior cingulate cortex, and orbital gyri being involved (28). Even if BPSD worsens due to frontal lobe damage, it may be possible to play musical instruments if motor and visuospatial functions are relatively preserved, as described below. Second, learning how to play a new accessible digital musical instrument may require motor skills, which can be attributed to the procedural, non-declarative forms of memory (29, 30). Non-declarative memory includes different forms of learning and memory abilities, including perceptual and motor skills involved in musical performance. The basal ganglia, cerebellum, and supplementary motor regions play a collective role in procedural memory (31, 32). As these areas are generally not affected by VaD, Ms. A. may have acquired and maintained the procedural memory skills required for playing a musical instrument. Third, visuoperceptual skills are required to read musical scores (26). Patients with VaD often have preserved visuo-constructive abilities because the posterior parietal regions are not significantly affected (33); this may have allowed Ms. A. to read the score and to play music on Cymis. VaD is associated with aphasia along with visuospatial and motor impairments, depending on the location of the affected area of the cerebrum, and symptoms tend to fluctuate everyday due to inadequate cerebral blood flow (33). However, in some cases, as in the patient in this study, it is possible for the person to play a musical instrument. Therefore, the preserved functions should be assessed before intervention, which should be tailored to the person's preserved functions (34).

The results of this study showed that even if BPSD continues to worsen, if the ability to play musical instruments is relatively preserved, it is possible to play accessible digital musical instruments in MT and to continue MT even in the later stages of dementia.

Our study had some limitations. We did not use the severe impairment battery (35) ideal test and did not examine the performedADLs (36) on a regular basis, similar to other neuropsychological tests. Moreover, we could not establish a pathological diagnosis for the patient. Finally, this was a case study; thus, the findings cannot be generalized.

In conclusion, this study showed that it is possible for people with late-stage VaD to learn to play a new accessible digital musical instrument and to continue to play it despite progressive BPSD with cognitive decline, which may have implications for the use of MT and music-based cognitive rehabilitation in persons with VaD.

## Data Availability Statement

The original contributions presented in the study are included in the article/supplementary material, further inquiries can be directed to the corresponding author.

## Ethics Statement

The studies involving human participants were reviewed and approved by the Research Safety and Ethics Committee of the Advanced Institute of Industrial Technology (AIIT). Written informed consent to participate in this study was provided by the patient's legal guardian/next of kin. Written informed consent was obtained from the patient's legal guardian/next of kin for the publication of any potentially identifiable images or data included in this article.

## Author Contributions

KT: conceived and designed the experiments and analyzed the data. EK: conducted the experiments. KT, EK, and KA: wrote the paper. RO and KA: contributed materials. KA: supervised and interpreted the data. All authors read and approved the final version of the article.

## Funding

This study was supported by JSPS KAKENHI (Grant Numbers: 17K17811, 20H04303, and 21K12194).

## Conflict of Interest

The authors declare that the research was conducted in the absence of any commercial or financial relationships that could be construed as a potential conflict of interest.

## Publisher's Note

All claims expressed in this article are solely those of the authors and do not necessarily represent those of their affiliated organizations, or those of the publisher, the editors and the reviewers. Any product that may be evaluated in this article, or claim that may be made by its manufacturer, is not guaranteed or endorsed by the publisher.
